# Incidental Computed Tomography Findings Among Traumatized Adults: A One-Year Analysis at a Trauma Center

**DOI:** 10.7759/cureus.51904

**Published:** 2024-01-08

**Authors:** Sawsan Sabiq, Abdulaziz Alzauir, Sarah A Alenizi

**Affiliations:** 1 Department of Nuclear Medicine, King Abdullah Medical City, Jeddah, SAU; 2 Department of Radiology, King Fahad Armed Forces Hospital, Jeddah, SAU

**Keywords:** incidentaloma, trauma, ct scan, findings, incidental

## Abstract

Background

Computed tomography (CT) for patients with trauma represents a widely accepted diagnostic method. Non-trauma-related incidentalomas or incidental findings are commonly depicted based on CT examination. Our study aimed to assess the frequency of incidental findings on CT scans among traumatized adult patients during one year at a trauma center.

Methods

We performed a retrospective case review of all adult patients triaged at the King Abdullah Medical Complex trauma service in Jeddah, Saudi Arabia, between 31 May 2022 and 30 May 2023. Patients under the age of 18 were excluded from the study. Patients who could not complete radiographic studies due to deterioration in condition, patients with missing CT scan reports, and transfer patients who had CT scans done at outside facilities were excluded. Demographic data, including age, sex, type of trauma, and type of CT, were recorded. All CT studies were reviewed for incidental findings.

Results

A total of 106 incidental findings were discovered in 99 patients. The rate of incidental findings for one year was 1.87%. The average age was 41.19 ± 17.90 years, with 73 (73.7%) male and 26 (26.3%) female patients. In trauma classifications, road traffic accidents were the most common (59.60%), followed by falls (33.33%), penetrating trauma (3.03%), and others. A high number of scans for the whole body (56.57%); face, brain, and cervix (13.13%); chest and abdomen/pelvis (11%); and spine (5.05%) had incidental findings. Genitourinary-related incidental findings were observed in 27.36% of patients, followed by craniospinal (16.98%), pulmonary (12.26%), hepatobiliary (9.43%), endocrine (9.43%), and musculoskeletal (5.66%).

Conclusion

In trauma centers, incidental findings are frequently discovered during CT imaging. In contrast, our center has a lower incidental finding rate. A whole-body CT scan yielded more incident findings than a selective one. The incidental findings are prevalent in the genitourinary system and higher in young male patients. It is important to properly document, communicate, and follow up on these incidental discoveries.

## Introduction

Trauma is defined generally as any violence or external force that causes physiological or psychological injury. Trauma systems establish cooperative ties between the local healthcare system and the emergency medical system or services to provide a comprehensive care approach for injured patients. A trauma system strives to ensure that persons who have been injured are treated properly, triaged, and moved to the right facility as soon as possible [1]. Traumatic injuries are a major public health concern around the world. On a global scale, injuries are predicted to overtake communicable diseases as the largest cause of disability-adjusted life years lost by 2020. Saudi Arabia is one of many developing countries that have seen a high rate of injury-related morbidity and mortality. Because Saudi Arabia is such a young country (40% of the population is under 19 years old), accidents can have a big impact on the country's health and prosperity. Traumatic injuries account for 22.6% of years of potential life lost in Saudi Arabia, according to the Global Burden of Disease Report [2].

An incidental finding is characterized by the identification of a new mass or lesion during imaging that was done for an unrelated purpose. The chance to identify an incidental finding could lead to the early detection of perilous diseases, such as cancer [3]. Over the last three decades, a rise in the use of cross-sectional imaging tests has resulted in a significant increase in the number of incidental findings discovered that are unrelated to the examination and clinical queries [4]. In the last two decades, the use of CT in injured patients has steadily increased. Rising accessibility, higher sensitivity to identify injury, physician-related factors, patient and family expectations, and, for some clinicians, fear of malpractice litigation due to a missed diagnosis are all possible explanations for the increased usage of sophisticated radiography [5,6].

The importance of incidental findings varies, ranging from minor lesions with no clinical significance to lesions that could have a significant influence on the trauma patient's health. Medically and legally, documentation of incidental findings and advice or referrals given to patients is required to demonstrate that qualified medical services and patient notice have been provided. Failure or delay in diagnosis can result in significant time loss and higher medical expenses and has been described as the most common medical mishap leading to malpractice cases. Patient communication and referrals based on incidental findings of significance and emergencies are critical because they demonstrate that care requirements have been met [7]. The rate of incidental findings in literature studies varies from 30% to 53% [8]. Our study aimed to determine the prevalence of incidental findings in CT among traumatic adult patients in a trauma center for one year.

## Materials and methods

Patients and settings

This study was a single-center, retrospective chart review conducted at the trauma center within King Abdullah Medical Complex in Jeddah, Saudi Arabia, spanning from May 31, 2022, to May 31, 2023. Patients were identified through the trauma registry maintained at the hospital over the preceding year, specifically those admitted via the emergency department. Ethical approval was obtained from the corresponding Institutional Review Board (IRB) - Ethics Committee of the hospital, with approval number A01378. All CT scan reports of trauma patients aged 18 years or older at the trauma center during the study period were included, excluding those under 18 years old, patients transferred from other facilities with CT scans completed elsewhere, and patients whose deteriorating condition prevented the completion of radiographic examinations.

Study tool and data collection

The review of CT scans was performed retrospectively by physicians and residents at the trauma center to identify any significant incidental findings. Data were collected using a pre-designed Excel data sheet. The initial section of the data sheet encompassed sociodemographic variables of the recruited participants, such as age and gender, while the primary segment focused on recording CT scan incidental findings.

Statistical analysis

For data analysis, Statistical Product and Service Solutions (SPSS, version 26; IBM SPSS Statistics for Windows, Armonk, NY) was utilized. The data entered into a standardized Excel sheet facilitated the calculation of incidental finding prevalence by dividing the number of incidental findings by the total number of CT scans performed during the previous year. Categorical and nominal variables were presented using numbers and percentages, whereas continuous variables were represented as mean and standard deviation.

## Results

From 31 May 2022 to 30 May 2023, 5,443 trauma patients presented to our trauma center and had a pan CT scan, or CT scan of the abdomen and pelvis, performed on initial presentation and had a report available in the EMR. Among these, 102 cases presented with positive incidental findings other than trauma-related. Ninety-nine patients who met the inclusion criteria were included in this present study, while three patients were excluded due to missing data on the type of trauma. The prevalence of patients found with incidental findings was 1.87%. A total of 106 incidental findings were found on non-contrast CT scan reports, and, of these, 8% of patients had multiple incidental findings. Table 1 lists the baseline characteristics of patients with incidental findings. There were 73 male and 26 female patients, with the mean age of all patients being 41.19 ± 17.90 years. The majority of the included cases were under the age group of 30-39 years (29.29%) old. In trauma classifications, road transport accidents were the most common (59.60%), followed by falls (33.33%), penetrating trauma (3.03%), and others. A high number of scans for the whole body (56.57%); face, brain, and cervix (13.13%); chest and abdomen/pelvis (11%); and spine (5.05%) had incidental findings. Additional diagnoses were recommended for 41 (41.41%) patients (Table 1).

**Table 1:** Baseline characteristics of patients CT: Computed tomography; SD: Standard deviation; RTA: Road traffic accidents

Variable	n (%)
Gender	n (%)
female	26 (26.3%)
male	73 (73.7%)
Age (Mean ± SD)	41.90 ± 17.90
Age category	
19-29	28 (28.28%)
30-39	29 (29.29%)
40-49	14 (14.14%)
>50	28 (28.28%)
Type of trauma	n (%)
RTA	59 (59.60%)
Fall	33 (33.33%)
Penetrating trauma	3 (3.03%)
Others	4 (4.04%)
CT performed	n (%)
PAN CT	56 (56.57%)
Facial, brain, and cervical	13 (13.13%)
Chest and abdominal/pelvis	11 (11.11%)
Spine	5 (5.05%)
Referred for additional diagnosis	41 (41.41%)

**Figure 1:**
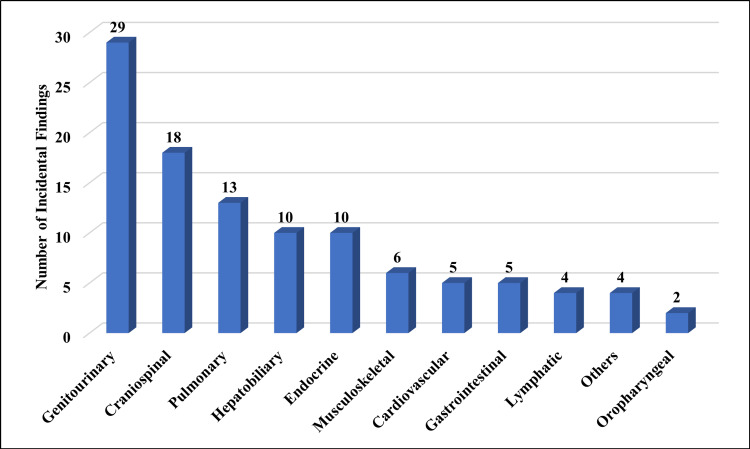
Distribution of incidental findings according to the body system

Genitourinary-related incidental findings were observed in 27.36% of patients, followed by craniospinal (16.98%), pulmonary (12.26%), hepatobiliary (9.43%), endocrine (9.43%), and musculoskeletal (5.66%). In less than 5% of the patients, gastrointestinal, lymphatic, and oropharyngeal incidental findings were found in the CT of trauma patients. The distribution of incidental findings according to body systems is listed in Table 2 and Figure 1.

**Table 2:** Distribution of incidental findings according to the body system

	n (%)
Genitourinary	29 (27.36%)
Craniospinal	18 (16.98%)
Pulmonary	13 (12.26%)
Hepatobiliary	10 (9.43%)
Endocrine	10 (9.43%)
Musculoskeletal	6 (5.66%)
Cardiovascular	5 (4.72%)
Gastrointestinal	5 (4.72%)
Lymphatic	4 (3.77%)
Oropharyngeal	2 (1.89%)
Others	4 (3.77%)

In the examination of a total of 99 trauma patients, incidental findings spanned a spectrum of body systems, illuminating the intricate nature of trauma-related assessments. This cohort revealed diverse incidental discoveries across various categories: neurological (n=18), pulmonary (n=13), oropharyngeal (n=2), musculoskeletal (n=6), cardiovascular (n=5), gastrointestinal (n=5), lymphatic (n=4), hepatobiliary (n=10), genitourinary (n=29), endocrine (n=10), and miscellaneous (n=4) anomalies. These incidental observations, encompassing cystic lesions, structural abnormalities, and organ-specific pathologies, underscore the imperative for comprehensive evaluations in trauma assessments.

Of note, patients exhibited a notable incidence of genitourinary-related anomalies, prominently featuring kidney stones. Gallbladder stones were the most frequently encountered within the hepatobiliary system. Although less frequent, findings across other systems - craniospinal, pulmonary, musculoskeletal, cardiovascular, gastrointestinal, lymphatic, endocrine, and miscellaneous categories - highlight the diverse range of incidental abnormalities encountered in trauma cases. These findings emphasize the necessity for thorough evaluation and clinical consideration in trauma patient care (Table 3).

**Table 3:** Incidental findings of 99 consecutive trauma patients according to the body system

Body system	Finding
Genitourinary (n=29)	Kidney stone (n=11), Renal cyst (n=6), Bilateral inguinal hernia (n=1), Non-obstructing hyperdense renal lesion (n=1), Left bifid rib (n=1), Solitary right kidney (n=1), Supra-renal cystic lesion (n=1), Enlarged prostate (n=1), Prostate cyst (n=2), Adnexal cyst (n=2), Corpus luteal cyst (n=1), Cystic lesion in the pelvis (n=1)
Craniospinal (n=18)	Retro cerebellar cystic lesion (n=2), Accessory ossicle of the anterior arch of C1 (n=1), Bilateral cysts within posterior lateral ventricle (n=1), Bilateral pars defect (n=1), Calcified meningioma (n=1), Fat-containing brain injury (n=1), Lytic lesion C4 (n=1), Middle cranial fossa cystic lesion (n=1), Paraspinal soft tissue (n=1), Partial empty sella (n=1), Posterior fossa cystic lesion (n=1), Right jugular foramen lesion (n=1), Right temporal arachnid cyst (n=1), Sacralization of L5 (n=1), Schmorl’s nodule (n=1), Signs of intra-cranial hypertension (n=1), Vertebral body haemangioma (n=1)
Pulmonary (n=13)	Anterior chest wall subcutaneous lipoma (n=1), Azygos lobe (n=1), Bilateral apical lung lesion (n=1), Emphysematous change in the right upper lung (n=1), Idiopathic Pulmonary Fibrosis (n=1), Left lower lobe consolidation (n=1), Left upper pulmonary mass (n=1), Lung subpleural emphysema (n=1), Pleural fat lesion (n=1), Paraseptal emphysema (n=1), Pulmonary hamartoma (n=1), Pulmonary nodule (n=1), Solitary pulmonary nodule (n=1)
Endocrine (n=10)	Thyroid nodule (n=3), Adrenal lesion (n=2), Bulky left adrenal gland (n=1), Enlarged thyroid with retrosternal extension (n=1), Left parotid cyst lesion (n=1), Adrenal myelolipoma (n=1)
Hepatobiliary (n=10)	Gallbladder stone (n=4), Hypodense liver lesion (n=2), Liver lesion (n=2), Common bile duct dilation (n=1), Liver cyst (n=1)
Musculoskeletal (n=6)	Arytenoid calcification (n=1), Benign lesion at the right iliac bone (n=1), Bilateral cervical rib (n=1), Cervical rib (n=1), H-shape vertebra (n=1), Non-aggressive bone island (n=1)
Cardiovascular (n=5)	Aneurysmal dilation of ascending aorta (n=1), Common origin of the left common carotid and brachiocephalic arteries (n=1), Left para-aortic soft tissue (n=1), Pericardial cyst (n=1), Right side aortic arch (n=1)
Gastrointestinal (n=5)	Fecalization of small bowel (n=1), Large bowel wall edema and fat infiltration (n=1), Sigmoid diverticulosis (n=1), Type 4 paraesophageal hernia (n=1), Mesenteric panniculitis (n=1)
Lymphatic (n=4)	Accessory spleen (n=1), Lymphocele (n=1), Splenic cystic lesion (n=1), Splenomegaly (n=1)
oropharyngeal (n=2)	Maxillary sinusitis (n=1), Tonsillar herniation (n=1)
Others (n=4)	

## Discussion

CT scans are frequently used to assess injuries in patients being evaluated for multisystem trauma. It is not uncommon for a single trauma patient to have a CT scan of the head, cervical spine, chest, abdomen, and pelvis as part of the workup. CT scans are an excellent modality for detecting traumatic injuries, as well as detecting other pathologies and conditions that may be present that are unrelated to the trauma. During normal workups, diagnostic imaging, and, more frequently, in emergency scenarios where imaging is performed after a traumatic event, incidental findings may be discovered. The issue then becomes how best to handle the additional information obtained. While some of these unexpected findings are harmless and do not require further investigation, others necessitate recurrent imaging and close monitoring by the patient's primary care physician.

Our study showed that the rate of incidental findings in retrospective chart review was 1.87% in our trauma patients. Unsurprisingly, whole-body CT scans yielded the majority of the findings. Genitourinary-related incidental abnormalities were the most often seen, followed by craniospinal, pulmonary, and hepatobiliary observations. The higher rate of road accidents increased the use of head or full-body CT scans and other radiological modalities in the emergency department. In our study, most of the patient's trauma happened due to road traffic accidents, and most of them were in the age group of 19-39 years old. The incidental finding frequency is considered higher among patients who undergo whole-body CT than in those who undergo selective CT. Consistent with our data, incidental findings in whole-body CT scans for severely injured patients are very prevalent [9,10]. However, the overall rate of incidental findings in trauma patients in this present study was considerably lower compared to other published literature (up to 75%) [7,9-12]. There were factors such as age, type of trauma, and type of CT that may be the reason for the lower rate. Similar to the prevalence, the spectrum of different body system-related incidental findings is different in the literature. Abdomen and pelvis region-related incidental findings were commonly reported in previous literature [8,13,14]. In our study, the most reported incidental findings in the body system were genitourinary. According to Hanna et al., about the body regions, incidental findings are most recognized in the ovaries, lungs, liver, and kidneys [15]. In another research conducted in 2011 for three months, there were 682 CT scans among 600 patients: 199 abdomen & pelvis, 405 head, and 78 thorax. A total of 348 incidental findings were documented in 33.4% of the scans, of which 9.8% were reported to patients in discharge paperwork [13]. A study carried out over six months in 2020 reported that, in 315 patients, there were 523 incidental findings (1.7 per patient); the most common were lung (17.5%), kidney (13%), and liver (11%) [16]. A study conducted by Grattan et al. [17] in 2019 reported that, of the 300 patients in the study, 26.3% were identified as having 83 distinct significant findings, with the greatest number, and 21.7% falling into the atherosclerosis or "other vascular’ category. Gallbladder pathologies of 14.5% were the next most common, with cysts or masses on various organs of 43.4%, collectively comprising the most remaining findings. The authors further demonstrated that at least 2.4% of findings were life-threatening and necessitated urgent treatment. As the use of whole-body CT for trauma increases, there is an associated increase in the detection of incidental findings. An additional contributory factor is the rising use of trauma CT in older patients in whom there is a higher background prevalence of undetected but significant pathology. In our study, most of the patients were below 40 years of age, and most traumas were due to road traffic accidents. This may be the reason for the lower prevalence of incidental findings compared with other studies.

Thirty percent of incidental findings were reported in a retrospective analysis of CT imaging used for trauma assessment at an urban level 1 trauma hospital in 2017. The abdomen and pelvis had the largest percentage of incidental findings, according to the study (61.7%) [18]. In this study, 70.9% had multiple incidental findings, with an average of three findings per patient. For 2.8% of incidental findings, follow-up was required, and for 0.7% of findings, admission and immediate intervention were requisite. In the discharge letter, incidental results were only reported in 1.4% of cases. During the study period of a case-control study, 197 (26%) of 692 trauma patients had incidental findings requiring follow-up. Overall, 91% of study patients with post-discharge physician follow-up had documented follow-up of the incidental finding. There was a significant improvement in the rate of follow-up in the study group compared to that of the control group (51% vs. 11%; p < 0.0001) [19]. Trauma patients frequently have unintentional findings. To ascertain the long-term outcomes of individuals with clinically important incidental findings, adequate recording and follow-up are therefore required [17].

van Vugt et al. demonstrated that incidental findings led to the diagnosis of previously unidentified malignancies among nine patients. Authors further commented that routine thoracoabdominal multidetector CT in the evaluation of trauma patients revealed a significant number of incidental findings [20]. One hundred and forty-three incidental findings in a study by Kelly et al. needed immediate examination. Following that, 24 occult neoplasms were diagnosed. Furthermore, 259 patients had additional diagnostic testing recommended [21]. In our study, 40 patients required additional diagnostics. It is viable to decide whether further follow-up or medication is required based on radiologic results [20]. Numerous investigations have noted a lack of follow-up and documentation for these incidental findings, which has important clinical and medico-legal repercussions. Poor documentation has been reported in the past, which, in our opinion, may be the result of concentrating more on trauma-related injuries than on these incidental discoveries, which are expected [9,22-24].

Incidental findings in trauma patients cause considerable discussion and concern over which patients require significant additional diagnosis, follow-up, and surveillance. This places heavy demands on subspecialties for their expertise, increasing their workload and having an impact on the delivery of healthcare services. Many incidental observations that occur during trauma management are not crucial for the initial trauma therapy but could be crucial for the patient's long-term health. Therefore, the practical applicability of these findings must be evaluated in light of the patient's current injuries as well as their potential future health. Patient survival and morbidity are increased and decreased, respectively, by early detection of incidental findings [22]. It must be considered that incidental findings found by a recent CT scan that is clarified and thoroughly documented would no longer need to be clarified at great expense in the future. Well-designed prospective cost-benefit and cost-effectiveness studies are required to effectively address this.

Limitations

Several limitations of this study deserve discussion. Firstly, our research was restricted to the frequency of incidental findings. Second, because just one trauma center participated, it is difficult to generalize the findings. Thirdly, it is restricted to retrospective data without follow-up details and the outcome of incidental findings in the hospital file.

## Conclusions

This study delves into the incidental findings identified in trauma patients undergoing CT scans, offering valuable insights into their prevalence, distribution, and implications within a trauma center setting. Despite a lower incidence rate compared to existing literature, this research underscores the significance of incidental findings, highlighting the diverse spectrum of anomalies across various body systems. Notably, genitourinary-related findings were prominent, particularly among younger male patients. The study emphasizes the crucial role of whole-body CT scans in not only detecting trauma-related injuries but also uncovering clinically relevant, unrelated pathologies. Moreover, this investigation's uniqueness lies in its exploration of incidental findings within a specific demographic and geographic context, prompting further exploration into the reasons behind the observed lower prevalence. Ultimately, this research advocates for standardized protocols to manage incidental findings, enhancing healthcare provider collaboration and establishing clear guidelines for optimal patient care in trauma centers.
